# Delayed graft function is associated with an increased rate of renal allograft rejection: A retrospective single center analysis

**DOI:** 10.1371/journal.pone.0199445

**Published:** 2018-06-21

**Authors:** Susanne Weber, Thomas Dienemann, Johannes Jacobi, Kai-Uwe Eckardt, Alexander Weidemann

**Affiliations:** 1 Medizinische Klinik 4, Nephrologie und Hypertensiologie, Universitätsklinikum Erlangen, Friedrich-Alexander-Universität Erlangen-Nürnberg (FAU), Erlangen, Germany; 2 Medizinische Klinik, Nephrologie und Internistische Intensivmedizin, Charite, Berlin, Germany; 3 Medizinische Klinik 1, Nephrologie, Transplantation und internistische Intensivmedizin, Krankenhaus Köln Merheim, Klinikum der Universität Witten-Herdecke, Cologne, Germany; Istituto Di Ricerche Farmacologiche Mario Negri, ITALY

## Abstract

**Introduction:**

The association of delayed graft function (DGF) and biopsy proven acute rejection (BPAR) of renal allografts is controversial. Borderline rejections comprise a major portion of biopsy results but the significance of such histologic changes is debated. The present study explores the impact of DGF on BPAR with a special emphasis on discriminating the effects of borderline rejection.

**Methods:**

Single center analysis of 417 deceased donor kidney recipients (age>18; transplantation date 1/2008–2/2015). Patients with primary non-function were excluded. DGF was defined as the need for dialysis within the first week after transplantation. Acute rejection was defined according to Banff criteria. Cox proportional hazards models were used to examine the relationship of DGF with BPAR within the first year.

**Results:**

No graft loss was observed during the first year after transplantation. DGF significantly associated with BPAR in the first year, irrespective of whether borderline rejections were included (HR 1.71, 95%CI 1.16,2.53) or excluded (HR 1.79, 95%CI 1.13,2.84).

**Conclusion:**

DGF is significantly associated with rejection—with or without borderline changes—within the first year.

## Introduction

Kidney transplantation represents the treatment of choice in patients with kidney failure. However, especially in the first weeks after transplantation, complications are not uncommon. Among these complications is delayed graft function (DGF), which is most commonly defined as the need for dialysis within the first week after transplantation [1]. Multiple studies have reported incidences of DGF in deceased donor kidney transplant recipients between 20% and 50% [2–6]. DGF can be viewed as a form of severe acute kidney injury (AKI) attributed to the transplant process. It is a result of donor and recipient factors as well as ex vivo storage and manipulation of the organ itself [4,7]. A cascade of events results in injury of endothelial as well as tubular cells [4]. These mechanisms are suspected to trigger an inflammatory response causing increased allograft immunogenicity, which may then lead to allograft rejection [8,9].

DGF has been shown to be a risk factor for chronic allograft dysfunction and allograft failure [3,9]. These associations may in part be explained by a higher incidence of acute rejections in kidneys affected by DGF [3,10]. Nevertheless, studies linking DGF to biopsy proven acute rejections (BPAR) have been inconsistent, presumably due to inhomogeneous study cohorts, varying definitions for DGF or different thresholds for dialysis [3,5,6,11–19]. Also, most of these studies were published more than a decade ago or included participants who were transplanted 20 years prior. A recent study demonstrated an independent association of DGF and BPAR in a Canadian single center cohort [20]. However, due to differences in waiting time, use of expanded criteria donors (ECD), and donors after cardiac death organs, these results may not be generalizable to European cohorts.

‘Borderline rejection’ is an ambiguous category in the current Banff classification. It reflects the difficulty of distinguishing inflammatory lesions from changes induced by injuries other than rejection and defines changes insufficient for a diagnosis of acute rejection. It may reflect early T-cell mediated rejection, but it may also merely resemble the histological correlate of acute kidney injury. A study that examined molecular changes using microarrays revealed that 67% of indication biopsies classified as ‘borderline’ in light microscopy were reassigned as ‘non-rejection like’ after molecular phenotyping [21]. To our knowledge no prior study investigating the association of DGF and BPAR has accounted for borderline biopsies in its analysis. Therefore, overestimating the numbers of ‘true’ rejections might also explain the inconsistent results.

The main objective of the present study was to explore the association of DGF and BPAR in the first year after transplantation, with special consideration of the impact of the histological diagnosis of borderline rejection.

## Methods

### Study participants

**Fig 1:**
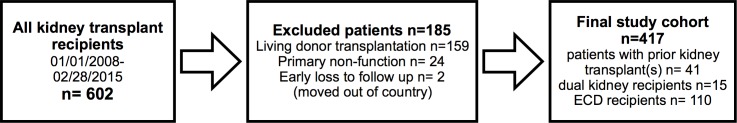
Schematic diagram. Study cohort.

Patients (aged >18 years) who received a deceased donor kidney allograft at the University Hospital Erlangen between January 1, 2008 and February 28, 2015 comprised the study population of this observational cohort study. The cohort comprised recipients of extended criteria donors, dual kidney recipients, and recipients who had a prior kidney transplant. We excluded living donor kidney transplant recipients and recipients with primary non-function. Furthermore we excluded patients who were lost to follow up within the first year (Fig 1). All transplant recipients were followed up at our outpatient clinic. The institutional review board of the University of Erlangen-Nürnberg approved the study protocol.

### Variables of interest

Patient medical records were reviewed by trained abstractors to obtain information on age, gender, body mass index (BMI), cause of end-stage renal disease, recipients of ECD organs, recipient diabetes status and history of coronary artery disease, number of prescribed anti-hypertensives, dialysis vintage, number of previous kidney transplantations, donor age, donor gender, donor BMI, donor diabetes status, donor history of hypertension, donor terminal kidney function, immunosuppressive induction regimen, type of calcineurin-inhibitor, and human leukocyte antigen (HLA) mismatch. In addition information was abstracted on warm ischemia time (WIT), cold ischemia time (CIT), peak panel reactive antibody (PRA) level, delayed or immediate graft function, number of dialysis sessions after transplantation, histological results of kidney biopsies, rejection therapy, number of biopsies, and renal function 12 months after transplantation. All patients were followed until the completion of the first post-transplant year.

### Immunosuppression/Post-transplant care

All patients received a triple immunosuppressive regimen, consisting of prednisone, mycophenolate and either tacrolimus or cyclosporine. In our center, unsensitized patients > 55 years and patients with reported pathological glucose tolerance without any immunological risk factors are treated with cyclosporine. All other patients are treated with tacrolimus. Recipients in ECD-program or with identified panel reactive antibodies > 5%, donor specific antibodies as well as prior kidney transplantation(s) receive anti-thymocyte globulin (ATG). The standard induction therapy is basiliximab.

Our center conducts protocol biopsies at 3 months after transplantation in patients with high PRA levels prior to transplantation, ECD organs or within the Eurotransplant acceptable mismatch program.

### Definitions

The exposure of interest, DGF, was defined as the need for ≥ 1 dialysis session during the first week after kidney transplantation. The clinical outcome in the present study was time to first BPAR including or excluding borderline rejection, which was defined according to Banff criteria on a renal biopsy pathology report. All pathology reports were read at our department of nephropathology. Only the first rejection of an individual patient was considered in the models. To reduce the possibility of a diagnostic bias, only indication biopsies were taken into account; none of the protocol-biopsies at 3 months after transplantation were included in the analysis. Indications for biopsies were at the discretion of the treating physician and included rising creatinine levels, persistent DGF or inability to achieve a better renal function within the first couple of months after transplantation. In context to DGF: we usually biopsy within 7 to 10 days after transplantation, if the patient is still dialysis dependent. Exact timing may be also influenced by other factors such as organ quality, immunology, cessation of urine output, and cold ischemia time. Renal function at 12 months after transplantation was assessed by the estimated glomerular filtration rate (eGFR) using the MDRD formula [22].

### Statistical analysis

STATA 14 (StataCorp LP, College Station, TX) was used for all statistical analyses. A p-value <0.05 was considered statistically significant and all tests were two tailed. Descriptive data are presented as means (SD) for continuous variables or frequencies for count data. Two-sided Student’s t-test, with adjustment for unequal variances where appropriate, or the Wilcoxon rank-sum test were used to examine differences between groups. Kaplan Meier curves and the log rank test were used to examine the cumulative incidence of time to first rejection in both groups.

Using a Cox proportional hazards model, the association of DGF with time to first BPAR, both including and excluding borderline rejection was tested. Despite full follow up a Cox proportional hazards model instead of a logistic regression model was chosen because of its ability to include the information of the exact timing of rejection [23–25]. Log-log plots and STATA’s “phtest” command were used to look for violations of the proportional hazards assumption. These models were adjusted for potential confounders using a sequential forward stepwise approach. These covariates were chosen on clinical judgment and literature review [16,26,27]. After careful assessment of correlation structures the following covariates were used in our models: age, gender, recipient BMI, PRA (as a categorical variable 0 vs. >0) dialysis vintage, cause of primary renal disease (coded as diabetic nephropathy vs. all others), donor age, donor sex, donor BMI, CIT, WIT, HLA-mismatch (A, B, DR), HLA-DR mismatch (coded as 0, 1, 2), tacrolimus as first line immunosuppressant (vs. cyclosporine), prior kidney transplantation (coded as 0, 1), induction therapy (none vs. IL-2 antagonist vs. depleting agent).

Due to a high correlation coefficient among donor hypertension and donor age, donor diabetes and donor age as well as ECD status and donor age, only donor age was included in the models. Owing to the relatively recent cohort and only very minor changes in our centers’ transplant protocol during the observation period, we did not include the variable “transplant year” into our model, which is sometimes done to adjust for unmeasured era effects. To assess robustness of our analysis we also tested an alternative definition of DGF (≥2 (vs ≥1) dialysis sessions in the first postoperative week).

A cut-off p-value of 0.2 was used for the univariate analysis. Linear regression was used to analyze determinants of renal function at the end of the observation period and its association with DGF. The linear regression model was adjusted for rejection, recipient age and sex, BMI, PRA, dialysis vintage, cause of end-stage renal disease, donor BMI, donor age, CIT, WIT, HLA-mismatch, HLA-DR mismatch, induction therapy, use of tacrolimus, prior transplant and terminal donor serum creatinine. We also tested for possible effect modification of DGF in combination with BPAR.

## Results

### Cohort characteristics

During the study period, 602 patients received a kidney allograft at our center. We excluded 159 living donor recipients, 24 patients who suffered from primary non-function and 2 patients who moved away from our center before completion of a 12 month follow up. The cohort comprised 417 patients. Out of these, 15 were recipients of a dual kidney transplantation, 41 underwent either the second, third or fourth kidney transplantation and 110 received a kidney from an ECD. (Fig 1) Most common causes of kidney failure in our cohort were glomerulonephritis (28.8%), followed by diabetes (12%), hypertension (11.3%) and ADPKD (11%). In 100 cases (23.9%) the cause for end-stage kidney disease was unknown. Seven Patients died with a functioning graft in their first year after transplantation. Cause of death was cardiac disease in 4 patients and unknown in 3 patients.

### Participant characteristics

The characteristics of the cohort stratified by DGF status are summarized in Table 1. In total, 143 patients (34.3%) developed DGF. There was no difference between the two groups regarding recipient and donor age, mean HLA or mean HLA-DR mismatch. Patients experiencing DGF were more often male and have had longer dialysis vintages before transplantation. The DGF group had higher terminal donor creatinine levels (115.05 μmol/l (± 88.5 μmol/l) vs 84.96 μmol/l (± 44.25 μmol/l), p <0.05) and included a higher percentage of patients with a previous transplant (14.0% vs. 7.7%, p <0.05). While WIT was significantly longer in our DGF group, CIT was not different. The majority of patients (75.5%) received tacrolimus as primary calcineurin inhibitor, 24.5% received cyclosporine. To 24.5% of all patients anti-thymocyte globulin was administered as induction therapy. Most patients (71.7%) received basiliximab and only 3.8% did not receive immunosuppressive induction. There was no difference in tacrolimus vs cyclosporine or anti-thymocyte globulin vs basiliximab use between the DGF and non-DGF groups.

**Table 1:** Participant characteristics.

	Final Study Cohort	Non- DGF	DGF	p-value
N	417	274	143	
Age (years)	53.5 (±12.7)	53.3 (±13)	53.8 (±12.1)	ns
Female	139 (33.3%)	104 (38%)	35 (24.5%)	**
BMI (kg/m2)	25.2 (±4)	25.1 (±4)	25.5 (±3.9)	ns
# of antihypertensive medications:0 ≤3 >3 ≥6	28 (6.7%) 261 (62.6%) 92 (22.1%) 32 (7.7%)	17 (6.2%) 175 (63.7%) 59 (21.5) 21 (7.7%)	11 (7.7%) 86 (60.1%) 33 (23.1%) 11 (7.7%)	ns
DM	88 (21.1%)	57 (20.8%)	38 (26.6%)	ns
CAD	95 (22.8%)	57 (20.8%)	38 (26.6%)	ns
Cause of Kidney Failure: Diabetes 2 vs. Other	50 (12%)	35 (12.8%)	15 (10.5%)	ns
Donor Age	52.9 (±16.2)	52.4 (±16.7)	53.8 (±15.3)	ns
Donor Female	213 (51.1%)	137 (50%)	76 (53.2%)	ns
Donor BMI (kg/m2)	27 (±5.9)	26.7 (±5.4)	27.5 (±6.6)	ns
Mean donor term. serum creatinine (μmol/l)	97.35 (±66.38)	84.96 (±44.25)	115.05 (±88.5)	*
Donor DM	51 (12.2%)	29(10.6%)	22 (15.4%)	ns
Donor hypertension	207 (49.6%)	132(48.2%)	75 (52.5%)	ns
PRA >0	51 (12.2%)	33 (12%)	18 (12.6%)	ns
Dialysis vintage (months)	70.6 (±46.3)	65.9 (±44.8)	79.7 (±48)	**
Mean HLA mismatch	2.8 (±1.7)	2.7 (1.7)	2.9 (±1.6)	ns
Mean HLA DR mismatch	0.9 (±0.8)	0.8 (0.8)	0.9 (±0.8)	ns
Mean CIT (h)	12.4 (±4.2)	12.2 (±0.3)	12.9 (±0.4)	ns
Mean WIT (min)	44.1 (±17.9)	41.4 (±15.4)	49.2 (±21.1)	***
CNI: Tacrolimus	315 (75.5%)	207 (75.5%)	108 (75.5%)	ns
Induction: anti-thymocyte globulin	102 (24.5%)	72 (26.3%)	30 (21%)	ns
Prior kidney transplantation	41 (9.8%)	21 (7.7%)	20 (14%)	*
Transplantation in ECD-program	110 (26.4%)	76 (27.7%)	34 (23.8%)	ns
Dual kidney transplantation	15 (3.6%)	10 (3.7%)	5 (3.5%)	ns

Variables either presented as mean (SD) or absolute values (relative frequencies)

* = p< 0.05

** = p<0.01

*** = p<0.001

BMI: body mass index, DM: diabetes mellitus, CAD: coronary artery disease, PRA: panel reactive antibody, HLA: human leukocyte antigen, CIT: cold ischemia time, WIT: warm ischemia time, CNI: calcineurin inhibitor, ECD: expanded criteria donor

### Rejection and renal function

**Fig 2:**
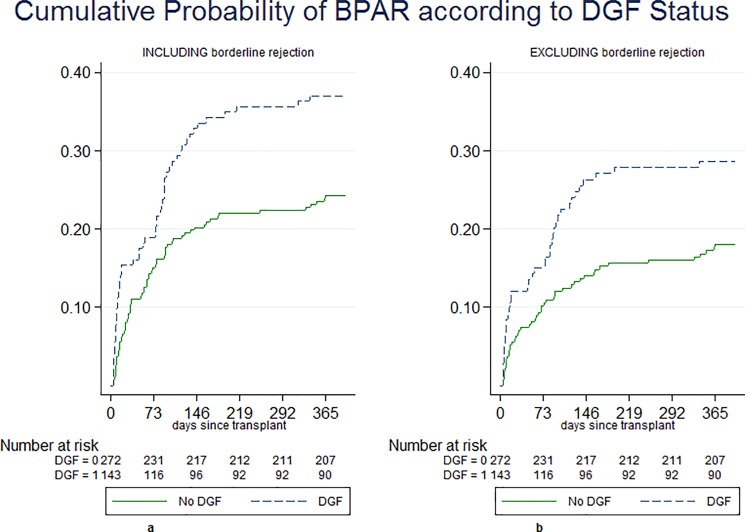
Kaplan Meier curves. Cumulative probability of BPAR according to DGF statusincluding borderline rejection (a) and .excluding borderline rejection (b).

Relative frequencies of BPAR including borderline rejections in year 1 were 24.8% in the non-DGF and 37.1% in the DGF group. In a total of 121 biopsy-proven rejections, we registered 100 T-cell mediated rejections and 21 antibody-mediated rejections. Patients in the DGF group developed more frequent T-cell mediated rejections (23.1%) compared with the non-DGF group (12.4%) (OR 1.98, 95%CI: 1.21, 3.24, p <0.01). Relative frequencies of BPAR excluding all ‘borderline rejections’ were 27.3% and 17.9% respectively (OR 1.72, 95%CI: 1.03, 2.85, p <0.05). (S1 Table) Kaplan-Meier curves of cumulative incidence of BPAR within the first year are presented in Fig 2A and 2B.

The unadjusted hazard ratio for developing acute rejection in the DGF group was 1.68 (95%CI: 1.17, 2.41, p <0.01). DGF remained an independent risk factor for BPAR after adjustment for multiple other variables in the stepwise cox regression models. Notably, hazard ratios did not change after expanding the model by covariates. In our full model (Model 5) recipient sex, dialysis vintage, CIT, WIT, cause of end-stage renal disease, recipient BMI, induction therapy, and terminal donor serum creatinine did not affect the association. Besides DGF, the hazard ratio for BPAR was significantly elevated for the categorical variable HLA-DR mismatch and prior transplant (DR mismatch = 1: HR 1.78, 95%CI: 1.13, 2.86, p = 0.01; DR mismatch = 2: HR 1.78, 95%CI: 1.05, 3.02, p <0.05; prior transplant: HR 2.09, 95%CI: 1.03, 4.27, p <0.05). PRA level and donor age missed the significance level (PRA level: HR: 1.81, 95%CI: 0.88, 3.72, p >0.05; donor age: HR 1.01, 95%CI: 0.99, 1.03, p >0.05). Protective factors for BPAR were older recipient age (HR 0.98, 95%CI: 0.96, 0.99, p <0.01) and tacrolimus use (HR 0.38, 95%CI: 0.25, 0.58, p<0.001). Multivariate cox models were calculated both with and without borderline rejections. (Tables 2 & 3)

**Table 2:** Multivariate table: HR for BPAR due to DGF.

Cox Model	Hazard ratio (95% CI)	p-value
Model 1	1.68 (1.17, 2.41)	**
Model 2	1.74 (1.2, 2.53)	**
Model 3	1.69 (1.16, 2.46)	**
Model 4	1.72 (1.18, 2.52)	**
Model 5	1.71 (1.16, 2.53)	**

Model 1: unadjusted model

Model 2: includes age, sex, BMI, PRA, dialysis vintage, cause of kidney failure

Model 3: includes covariates from Model 1 + donor age, donor BMI, donor sex, donor diabetes

Model 4: includes covariates from Models 1&2 + CIT, HLA DR mismatch, tacrolimus use, re-transplant status

Model 5: includes covariates from Model 1&2&3 + WIT, terminal donor serum creatinine, induction therapy

** = p<0.01

**Table 3:** Multivariate table: HR for BPAR excluding borderline rejections due to DGF.

Cox Model	Hazard ratio (95% CI)	P-value
Model 1	1.74 (1.13, 2.65)	*
Model 2	1.8 (1.16, 2.79)	**
Model 3	1.9 (1.15, 2.77)	*
Model 4	1.77 (1.13, 2.77)	*
Model 5	1.79 (1.13, 2.84)	*

Model 1: unadjusted model

Model 2: includes age, sex, BMI, PRA, dialysis vintage, cause of kidney failure

Model 3: includes covariates from Model 1 + donor age, donor BMI, donor sex, donor diabetes

Model 4: includes covariates from Models 1&2 + CIT, HLA DR mismatch, tacrolimus use, re-transplant status

Model 5: includes covariates from Model 1&2&3 + WIT, terminal donor serum creatinine, induction therapy

* = p< 0.05

** = p<0.01

When excluding borderline rejection, the unadjusted HR for developing BPAR was 1.74 (95%CI: 1.13, 2.65, p <0.05). The HR was robust after expanding the model with the earlier mentioned covariates (HR full model 1.79, 95%CI: 1.13, 2.84, p <0.05). HR for BPAR excluding borderline rejection was also significantly higher for HLA-DR mismatch (DR mismatch = 1: HR 1.83, 95%CI: 1.07, 3.14, p <0.05; HLA-DR mismatch = 2: HR 2.21, 95%CI: 1.19, 4.12, p <0.05). Use of tacrolimus (HR 0.4, 95%CI: 0.24, 0.66, p <0.001) and older recipient age (HR 0.77, 95%CI: 0.63, 0.95, p <0.05) were protective factors. Sensitivity analyses were conducted defining DGF as the need for at least 2 dialysis sessions. Of 143 patients undergoing post-transplant dialysis treatment, 111 (77.6%) required more than one session, of whom 41 (36.9%) experienced BPAR. After altering the definition for DGF, the hazard for BPAR was still significantly higher for DGF compared to non-DGF patients (HR 1.56, 95%CI: 1.03, 2.36, p <0.05). However, for this definition of DGF, the association between DGF and BPAR did not reach significance after exclusion of borderline rejections (HR 1.40, 95%CI: 0.85, 2.20, p >0.05). (S2 Table)

The overall number of biopsies in the first year was higher in the DGF group (non-DGF $\bar{x}$ 2.17 SD = 1.72; DGF $\bar{x}$ 2.50 SD = 1.94). This difference was not significant (p >0.05). (S1 Table, S1 Fig)

There was a significant difference in renal function at the end of the observation period. Mean eGFR in the DGF group was at 44.8 (± 4.02) ml/min and 46.6 (±2.46) in the non-DGF group. A linear regression model with eGFR at one year as the outcome showed no association between DGF and the outcome. Our model identified BPAR (Coefficient (c):

-5.01 ml/min, 95%CI: -8.18, -1.84) and donor age per decade (c: -2.38 ml/min, 95%CI:
-3.62, -1.13) as significant factors for a reduction in eGFR at one year. (Table 4) No effect modification was detected when using DGF*BPAR as an interaction term in the model.

**Table 4:** Multivariate table: Causes for decrease in eGFR at one year.

Linear Regression Model	Coefficient (95% CI)	P-value
DGF	0.06 (-2.91, 3.05)	ns
BPAR	-5.01 (-8.18, -1.84)	**
Donor age (per decade)	-2.38 (-3.62, -1.13)	***

Coefficient for eGFR in ml/min (MDRD) 1 year post transplantation

ns = not significant

** = p< 0.01

*** = p<0.001

## Discussion

In the present study the hazard ratio for developing BPAR within the first year after transplantation was 71% higher in the DGF group. This association was independent of covariates related to DGF in our cohort as well as covariates that were reported in previous studies [28,29]. Despite a probable biological link, previous studies could not uniformly demonstrate this association. These studies either used surrogate definitions for DGF or for the outcome of acute rejection, included inhomogeneous study cohorts or did not use multivariate modeling [19]. Our findings are consistent with the results from a very recent study by a Canadian group [20]. However, in this carefully executed study, the authors did not take into account WIT, choice of induction therapy and did not report on the number of biopsies in each group. Since WIT and induction therapy have both been shown to be associated with DGF, BPAR and transplant function [30–32] our data significantly contributes to the understanding of the association of DGF and BPAR as these are included in the present analysis.

An important difference of this study compared to previous studies is the thorough analysis of the contribution of borderline rejection. We believe that this is a critical distinction since biopsies labeled ‘borderline’ may reflect either early T-cell mediated rejection (which could advance to more severe inflammatory lesions), or the morphological correlate of AKI [21]. However, most of borderline changes without therapy do not progress to acute rejection [33] or lead to a deterioration of renal function after 2 years [34]. The course of the graft seems to depend on clinically manifest borderline changes (i.e. increased creatinine) which show immunity-related molecular changes [35]. Subclinical, histological borderline changes detected in protocol biopsies–at least on a molecular level–seem to reflect primarily the injury–repair response [35,36], and have been shown to be greater in organs with DGF. Thus, by excluding borderline rejections, the sensitivity of our analysis linking DGF to BPAR is increased: the inclusion of changes not progressing to rejection would inadvertently bias results away from the null hypothesis leading to an over-diagnosis of BPAR.

Our results are supported by the implementation of using different definitions of DGF, as these are not uniform in the literature [1]. The most commonly used definition (≥1 dialysis in the first week) encompasses patients with a great range of ischemic injury. The reason for dialysis can be somewhat arbitrary and therefore lead to possible distortion of the definition of DGF, which has been shown to impact the results of previous studies [1,15]. When using a different cutoff for the number of dialysis sessions in the first week (≥ 2 dialysis), the association between DGF and BPAR remained robust. However, when using the altered definition of DGF, in combination with the exclusion of borderline rejections, the significance level was barely missed. This may have been a sample size problem, as the number of patients with DGF dropped by almost 25% and the HR was still well above 1 with an asymmetric 95% CI ranging from 0.85 to 2.20.

After a 12 months follow-up period, patients in the DGF group had a lower eGFR compared to those with immediate allograft function. This was a result from the higher incidence of BPAR in the DGF group. The difference was statistically significant, however the absolute difference of 1.8 ml/min is, from our point of view, not clinically meaningful. Two single center studies on DGF, where the outcome was graft survival, reported similar results [19,37]. A recent registry study, analyzing more than 28.000 mate kidney transplants, has found an increased risk of graft failure in kidneys with DGF within the first year irrespective of BPAR [18]. Since we did not observe any graft losses within the first year (except for the patients with primary non-function who were excluded), no conclusion can be drawn whether DGF influenced graft survival.

Our study has several limitations. The most important caveat in any study of such kind is the possibility of a diagnostic bias from an unbalanced number of biopsies in the two groups. The overall number of biopsies in the first year was numerically higher in the DGF group. This difference was not statistically significant and was presumably caused by a greater number of repeat biopsies in the DGF group. In order to further reduce the risk of imbalance, we only considered results from indication biopsies for our analysis. The dataset lacked information on HLA- and donor-specific antibodies (DSA) at baseline and during follow up. We acknowledge that PRA is inferior to DSA regarding the risk for the development of an antibody mediated rejection (ABMR). However, the assessment whether or not patients with DGF have a predisposition to develop such antibodies was not our primary focus and incidence of T-cell mediated rejection outnumbered ABMR in our population. Nevertheless, due to the lack of HLA testing our data does not allow the conclusion of the association of DGF and ABMR. Additionally, information on immunosuppressant levels was not available. Although it is generally accepted that optimal calcineurin-inhibitor target concentrations reduce the risk for acute rejection, the effect is not always clearly seen in epidemiologic studies due to the interval between the beginning of a rejection episode and the ascertainment of the immunosuppressant level, as well as for suspected non-calcineurin driven mechanisms [38,39]. Generalizability of our results may be reduced because it represents a Caucasian only, single center study population. Furthermore, as with any retrospective study there may be residual confounding despite multivariate adjustment. The short follow up time prohibited any inference on the long-term impact of DGF on graft function. We did not have the exact biopsy-dates, which prohibited us from adjusting for the number of biopsies until the occurrence of first rejection.

One of the strengths of this study is the high granularity of our cohort, which allowed us to include important covariates such as WIT, which we feel is underreported in the literature. In our non-DGF group no patient required dialysis treatment during the observation period and we had to exclude only 2 patients due to loss of follow up. Moreover, since all biopsies were analyzed at the same nephropathology lab, we have a high level of consistency of the histological diagnoses compared to registry data, which is of special relevance for our aim of identifying the contribution of borderline rejection.

In summary our results confirm the importance of DGF as an independent risk factor for BPAR. The assumption is strengthened by omitting borderline rejections, which could have inflated the association due to an overestimation of rejection episodes in previous studies. Despite improvements in organ allocation and advances in immunosuppressive therapy, the incidence of DGF has increased since the 1990s and may still rise in the future due to the acceptance of more marginal organs [6,40–43]. A close surveillance of patients with DGF is warranted to mitigate potentially adverse effects in this group.

## Supporting information

S1 Table Study outcome due to DGF status (mean and absolute values). (PDF)

S2 Table Multivariate table: HR (Model 5) for BPAR due to DGF (≥ 2 dialysis post-transplant). (PDF)

S1 Fig Bar diagram. Relative frequency of biopsies in each group.
(PDF)

## Abbreviations

ABMR: antibody mediated rejection
AKI: acute kidney injury
ATG: anti-thymocyte globulin
BMI: body mass index
BPAR: biopsy proven acute rejection
CIT: cold ischemia time
C: coefficient
DGF: delayed graft function
ECD: expanded criteria donor
HLA: human leukocyte antigen
HR: hazard ratio
OR: odds ratio
PRA: panel reactive antibody
WIT: warm ischemia time
