# Supplementary material for: Delayed graft function is associated with an increased rate of renal allograft rejection: A retrospective single center analysis
Source: PLoS One. 2018 Jun 21;13(6):e0199445. doi: 10.1371/journal.pone.0199445 (PMC6013231; doi:10.1371/journal.pone.0199445)
Supplement: S1 Table — (PDF) [file pone.0199445.s001.pdf]

## Table 5. Study outcome due to DGF status (mean and absolute values).

| | Non-DGF (n=274) | DGF (n=143) | P-value |
|---|---|---|---|
| **Mean # of total biopsies within first year** | 2.17 (±1.72) | 2.50 (±1.94) | ns |
| **Mean eGFR at 12 months (ml/m$^2$)** | 46.6 (±1.49) | 44.8 (±0.34) | *** |
| **Acute Rejection:** | 68 (24.8%) | 53 (37.1%) | ** |
| **Borderline** | 19 (27.4%) | 14 (26.4%) | |
| **Banff Ia** | 14 (20.5%) | 16 (30.1%) | |
| **Banff Ib** | 4 (5.8%) | 3 (5.6%) | |
| **Banff IIa** | 16 (23.5%) | 12 (22.6%) | |
| **Banff III** | 0 | 2 (3.7%) | |
| **Humoral** | 15 (20.0 %) | 6 (11.3%) | |

Variables either presented as mean (SD) or absolute values (relative frequencies), * = p< 0.05 ; ** = p<0.01 ; *** = p<0.001
