# Supplementary material for: Delayed graft function is associated with an increased rate of renal allograft rejection: A retrospective single center analysis
Source: PLoS One. 2018 Jun 21;13(6):e0199445. doi: 10.1371/journal.pone.0199445 (PMC6013231; doi:10.1371/journal.pone.0199445)
Supplement: S2 Table — (PDF) [file pone.0199445.s002.pdf]

Table 6. Multivariate Table: HR (Model 5) for BPAR due to DGF (≥ 2 dialysis post-transplant).

DGF defined by ≥ 2 dialysis treatments post-transplant	Non-DGF (n=306)	DGF (n=111)	Hazard ratio (95% CI)	P-value
**BPAR with borderline rejection**	80 (26.1%)	41 (36.9%)	1.56 (1.03, 2.36)	*
**BPAR without borderline rejection**	60 (20.9%)	28 (25.2%)	1.40 (0.85, 2.2)	ns

Multivariate Table: HR (Model 5) for BPAR due to DGF (≥ 2 dialysis post-transplant)
Outcome by group (DGF definition ≥ 2 dialysis treatments required after transplantation)
* = p< 0.05 ; ** = p<0.01 ; *** = p<0.001
