# Supplementary figures and images for: Delayed graft function is associated with an increased rate of renal allograft rejection: A retrospective single center analysis
Source: PLoS One. 2018 Jun 21;13(6):e0199445. doi: 10.1371/journal.pone.0199445 (PMC6013231; doi:10.1371/journal.pone.0199445)

Relative Frequency of Biopsies in each Group

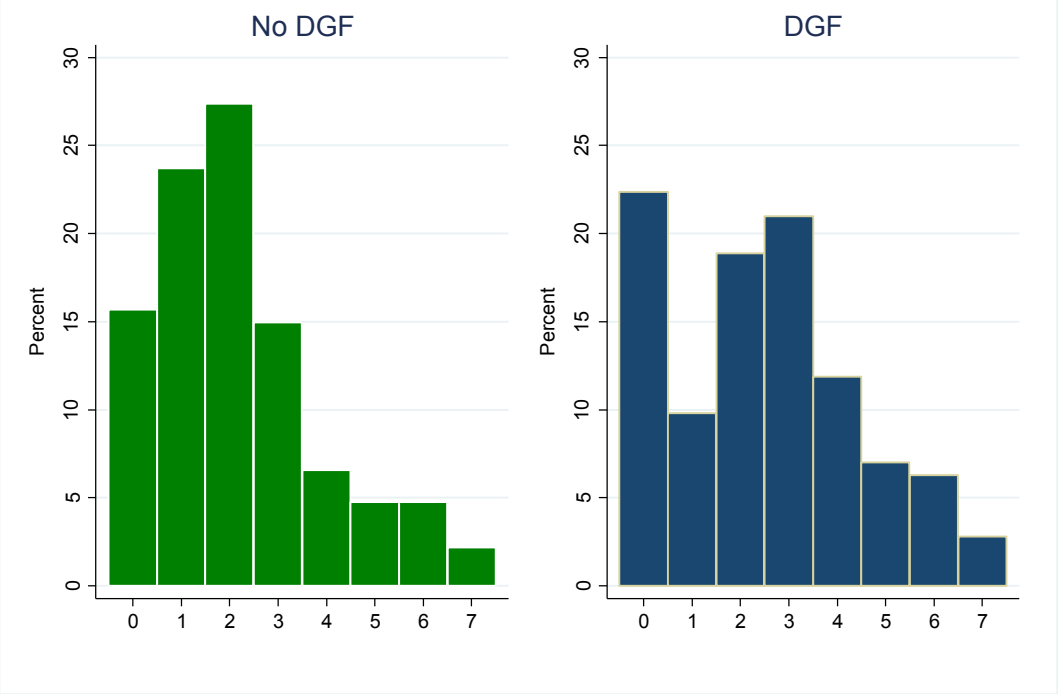

Supplement: S1 Fig — Relative frequency of biopsies in each group. (PDF) [file pone.0199445.s003.pdf]
